# A study of wound repair in *Dictyostelium* cells by using novel laserporation

**DOI:** 10.1038/s41598-018-26337-0

**Published:** 2018-05-22

**Authors:** Mst. Shaela Pervin, Go Itoh, Md. Shahabe Uddin Talukder, Koushiro Fujimoto, Yusuke V. Morimoto, Masamitsu Tanaka, Masahiro Ueda, Shigehiko Yumura

**Affiliations:** 1 Department of Functional Molecular Biology, Graduate School of Medicine, Yamaguchi University, Yamaguchi, 753-8512 Japan; 2 Department of Molecular Medicine and Biochemistry, Akita University Graduate School of Medicine, Akita, 010-8543 Japan; 3 Institute of Food and Radiation Biology, Atomic Energy Research Establishment, Savar, GPO Box 3787, Dhaka, 1000 Bangladesh; 4 Department of Bioscience and Bioinformatics, Faculty of Computer Science and Systems Engineering, Kyushu Institute of Technology, 680-4 Kawazu, Iizuka, Fukuoka, 820-8502 Japan; 5 Quantitative Biology Center (QBiC), RIKEN, 6-2-3 Furuedai, Suita, Osaka, 565-0871 Japan; 6 Graduate School of Frontier Biosciences, Osaka University, Osaka, 565-0871 Japan

## Abstract

We examined the mechanism of cell membrane repair in *Dictyostelium* cells by using a novel laser-based cell poration method. The dynamics of wound pores opening and closing were characterized by live imaging of fluorescent cell membrane proteins, influx of fluorescent dye, and Ca^2+^ imaging. The wound closed within 2–4 sec, depending on the wound size. Cells could tolerate a wound size of less than 2.0 µm. In the absence of Ca^2+^ in the external medium, the wound pore did not close and cells ruptured. The release of Ca^2+^ from intracellular stores also contributed to the elevation of cytoplasmic Ca^2+^ but not to wound repair. Annexin C1 immediately accumulated at the wound site depending on the external Ca^2+^ concentration, and annexin C1 knockout cells had a defect in wound repair, but it was not essential. *Dictyostelium* cells were able to respond to multiple repeated wounds with the same time courses, in contrast to previous reports showing that the first wound accelerates the second wound repair in fibroblasts.

## Introduction

The cell membrane functions as a barrier between the extra and intracellular spaces. However, cells are consistently subjected to wounding by physical or chemical damages from the external environment. In our bodies, the stretch and contraction in muscle tissue and hydrostatic pressure in the cardiovascular system frequently injure the cell membrane. Wounded cell membrane loses its barrier function, resulting in an influx of undesirable substances into the cell as well as loss of cytoplasm. However, the cells have an ability to repair the wounded cell membrane. Defects in cell membrane repair may cause muscular dystrophy^1,2^, diabetes^3^, vitamin deficiencies^4^ and inflammatory myopathy^5^. Thus, like DNA repair, wound repair is a physiologically vital phenomenon for living cells. In addition, many methods for introducing extracellular substances into cells, including microinjection and electroporation, rely on cellular wound repair.

The molecular mechanism of wound repair has been studied in different model organisms such as mammalian cells, amphibian eggs, echinoderm eggs, fruit flies, amoebae, and budding yeast^6–11^. A common feature among them is that Ca^2+^ in the external medium is essential for wound repair. The cytoplasmic concentration of Ca^2+^ is maintained at a sub-micromolar level. The entry of elevated levels of Ca^2+^ is harmful for cell survival and finally results in apoptosis or cell death. The entry of Ca^2+^ is considered to mediate the recruitment of vesicles to reseal the wound pore^12,13^. The release of Ca^2+^ from intracellular stores may also contribute to wound repair^14^. The cytoplasmic vesicles fuse by exocytosis with the cell membrane, either directly as single vesicles or as a patch formed by the homotypic fusion of intracellular vesicles^15^. The removal of wounded membrane by endocytosis or shedding also contributes to the wound repair, depending on the wound size^16–18^. Cortical actin cytoskeleton is also rearranged during wound repair^6,19,20^. Annexin, a Ca^2+^-dependent membrane scaffold protein family, which binds to negatively charged membrane phospholipids, such as phosphatidylserine, in a Ca^2+^-dependent manner, has been implicated in muscle cell membrane repair^21–23^. Annexin also accumulates at the wound sites in other cells, and dysfunction of annexin inhibits the resealing process^24–27^. It has been reported that several other proteins and signals also accumulate as the wound repair machinery^19,28–30^. Despite several models proposed for the molecular mechanism of wound repair^17,31^, the detailed molecular mechanism remains unclear. One reason is that the involved molecular species differ among different species of cells. Therefore, additional cell types must be examined for comparison.

We previously examined wound repair in *Dictyostelium* cells by partially cutting them with a microneedle^32^. Even when a cell is separated into two portions by cutting, the fragment containing a nucleus can exhibit normal chemotactic movement^33,34^, suggesting that *Dictyostelium* cells have a wound repair system. However, it is difficult to regulate the wound size and exact timing of the wounding by manually using a microneedle. We recently invented a novel laser-based cell poration method to introduce foreign molecules into single cells^35^. Only a short-term exposure of a low power laser is sufficient for this laserporation method, although a high-power pulse laser or long-term exposure of low-power laser had been used for other cells in previous experiments. In addition, our laserporation only wounds the cell membrane, although the previous laserporation may also wound the inside of the cell.

Previous studies using other organisms have revealed that Ca^2+^ in the external medium is necessary for wound repair^11,36^. However, there are few reports addressing basic questions of whether Ca^2+^ certainly enters through the wound pore, whether the larger wounds lead to the greater influx of Ca^2+^, when the wound functionally closes, and when the wound becomes even with the uninjured membrane. Unlike poking or cutting with a microneedle as used in previous studies, now we can precisely regulate the wound size using our laserporation method. By using this powerful method, we observed, for the first time, the dynamics of wound pore opening and closing, Ca^2+^ influx, and annexins in *Dictyostelium* cells. These results show that *Dictyostelium* cells can be an excellent new model organism for wound repair research.

## Results

### A new application of laserporation to wound the cell membrane

**Figure 1:**
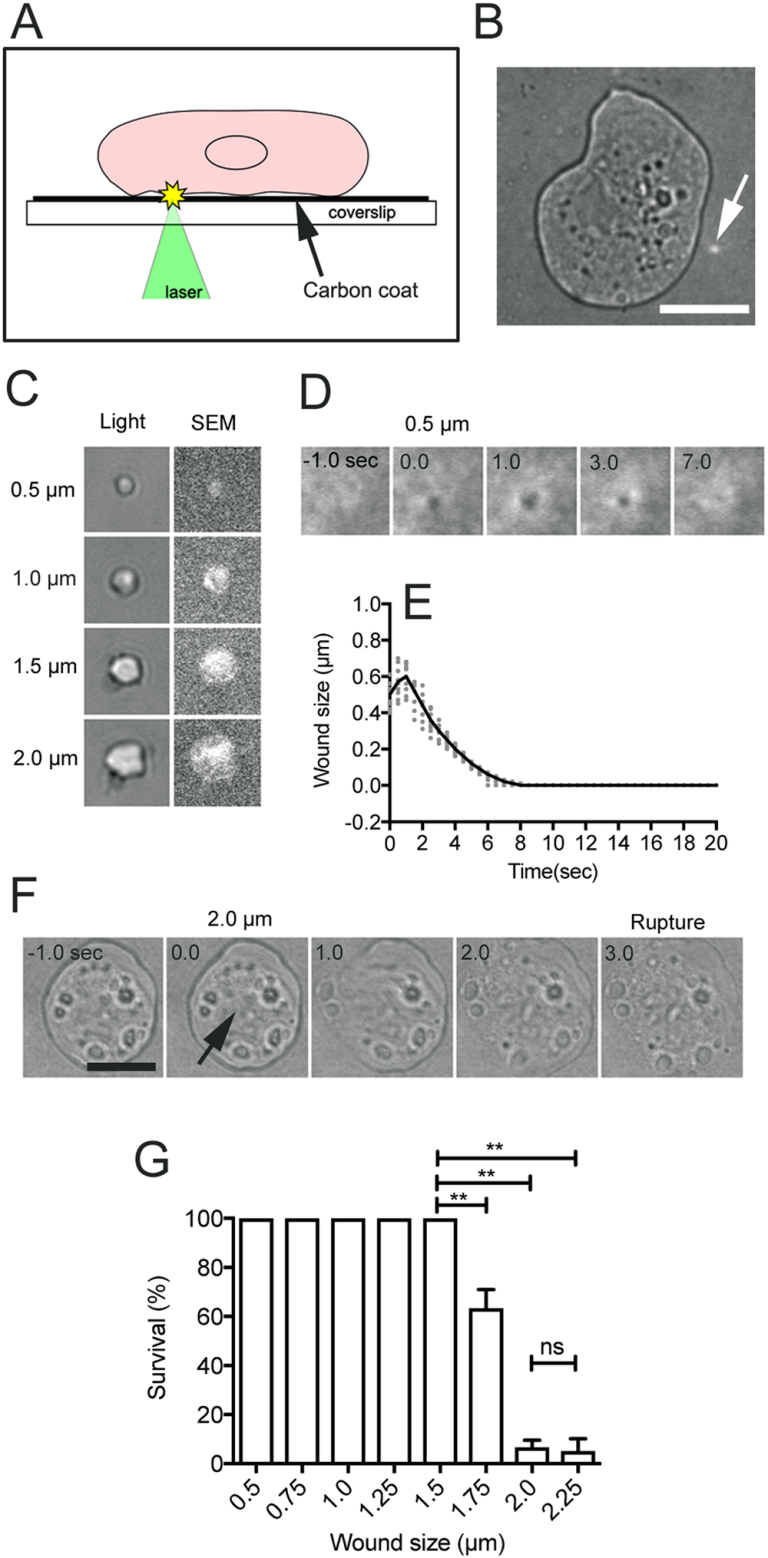
A new application of laserporation to wound the cell membrane. (**A**) After cells were placed on a carbon-coated coverslip, a laser beam was focused on a small local spot beneath a single cell under a TIRF microscope. The absorbed energy of the laser beam by the carbon created a small pore in the cell membrane that was attached to the carbon coat. (**B**) When the laser beam was focused on the surface of coated coverslip, the carbon coat was peeled off, appearing as a small white spot (arrow). (**C**) White spots of different sizes (0.5, 1.0, 1.5, and 2.0 µm) on the carbon coat observed by light and scanning electron microscopy. (**D**) When the cell expressing GFP-cAR1 was wounded by laserporation, a black spot appeared in the cell membrane. The black spot transiently expanded, then shrank, and finally closed. (**E**) Time course of the diameter of the black spot after the laserporation. The solid line is an averaged line of 10 experiments (gray dots). (**F**) A typical sequence of bright field images of cell rupture with a 2 µm wound (arrow). (**G**) Survival rate of cells with different laser spot sizes. The number of cells that ruptured within 5 min after laserporation was counted. Data are presented as mean ± SD (n = 20, 3 experiments, each). **P ≤ 0.0001; ns, not significant, P > 0.05. Bars, 10 µm.

To wound the cell membrane of *Dictyostelium* cells, we applied a laser-based cell poration method, which we recently invented for the introduction of foreign molecules into live cells^35^. After cells were placed on a coverslip that had been coated with carbon by vapor deposition, a laser beam was focused on a small local spot beneath a single cell under a total internal reflection fluorescence (TIRF) microscope. The absorbed energy of the laser beam by the carbon made a small pore in the cell membrane that was attached to the carbon coat (Fig. 1A). It was easy to focus the laser spot on the surface of the coverslip; when the laser beam was focused on the surface of the coated coverslip, the carbon coat was peeled off, appearing as a small white spot (arrow in Fig. 1B). Figure 1C shows white spots of different sizes (0.5, 1.0, 1.5, and 2.0 µm) on the carbon coat, which were observed by light and scanning electron microscopy. The wound size could be regulated by changing the pinhole size placed in the pathway of the wound laser or changing the laser power. After the focus was set on the surface of the coverslip in this way, the laser beam was applied to the cells attached to the coverslip. Because the laser power used for the actual wound experiments was set much lower, the carbon coat was not peeled off. Thus, as described later, multiple wounding experiments were also possible at the same site.

To visualize the wound pore in the cell membrane, the laser was applied to the cells expressing GFP-cAR1 (cAMP receptor), as a marker of membrane protein. Immediately after the laser application, a black spot appeared at the laser-applied position (Fig. 1D). If the coverslip was not coated with carbon, such black spots did not appear, indicating that the black spot was not due to photobleaching by the laser beam. The size of the black spot was the same as that of the white spot (0.5 µm) in the carbon coat shown in Fig. 1C. Figure 1E shows time course of the size of the black spot after the laser application. The black spot transiently expanded slightly, then shrank, and finally closed within approximately 8 sec (7.41 ± 0.95 sec, n = 10). If the size of the laser spot increased up to 2 µm, most of the cells immediately ruptured (Fig. 1F). Figure 1G shows the survival rate with different sizes of the laser spot. The cells could repair wounds with a size less than 2.0 µm.

### Influx of fluorescent dye from the wound pore

**Figure 2:**
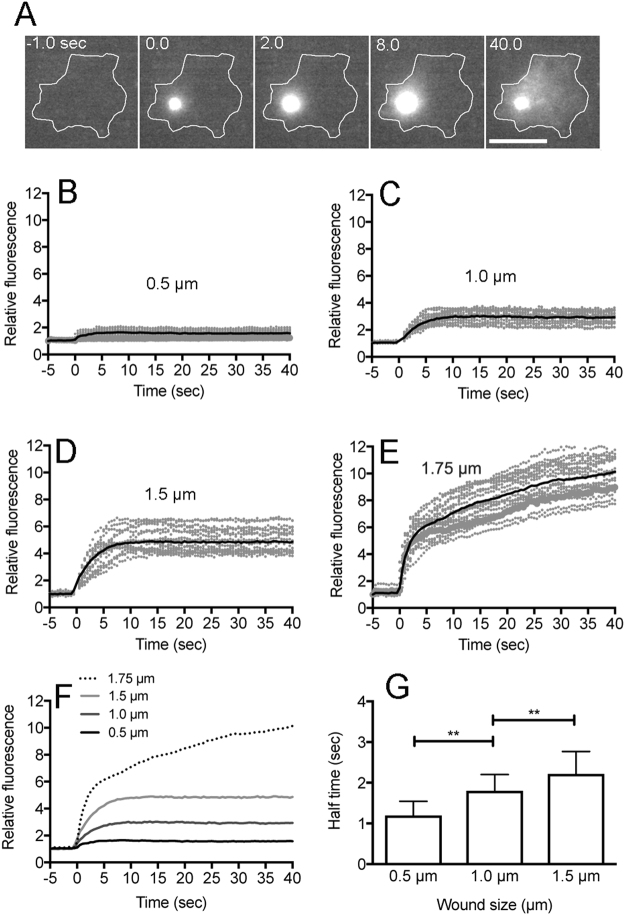
Influx of fluorescent dye from the wound pore. Wound experiments were performed in BSS including propidium iodide (PI). (**A**) A typical sequence of fluorescence images of PI influx after laserporation. The wound laser beam was applied at 0 time and the duration was set at 8 msec. Note that the fluorescence began to increase at the wound site and spread over the cytoplasm. (**B**–**E**) Time courses of PI influx with different wound sizes (0.5, 1.0, 1.5, and 1.75 µm, n = 27, each). (**F**) A comparison of time courses of PI influx in 4 different wound sizes (0.5, 1.0, 1.5, and 1.75 µm). (**G**) Half time of closing with different wound sizes. The half time was examined by curve fitting as described in Methods section. The half time was 1.19 ± 0.34 sec with a 0.5 µm wound, 1.80 ± 0.40 sec with a 1.0 µm wound, and 2.22 ± 0.55 sec with a 1.5 µm wound after the laserporation, respectively (n = 27, each). Data are presented as mean ± SD. **P ≤ 0.0001. Bar, 10 µm.

If the laser beam makes an actual pore in the cell membrane, the fluorescent dye in the external medium will enter the cell through the pore. Propidium iodide (PI), which emits red fluorescence when it binds to RNA and DNA, was included in the external medium BSS, a physiological saline that contains 3 mM Ca^2+^. Figure 2A shows a typical fluorescence microscopy of PI influx from the wound pore. The fluorescence began to increase at the wound site and spread over the cytoplasm. Figure 2B–E show time courses of PI influx with different wound sizes (0.5, 1.0, 1.5, and 1.75 µm). Figure 2F shows a comparison of the time course of PI influx with 4 different wound sizes. Increased fluorescence was observed with larger wound sizes. The fluorescence intensity of PI increased immediately after wounding and ceased to increase within a few seconds, which represents the time of wound closing. Figure 2G shows the half time of closing time with different wound sizes. With a larger wound size (1.75 µm), the influx of PI did not cease for a long time (Fig. 2E). Based on these experiments, we confirmed that the laserporation reliably creates a wound pore in the cell membrane.

### Ca^2+^ influx from the wound pore

**Figure 3:**
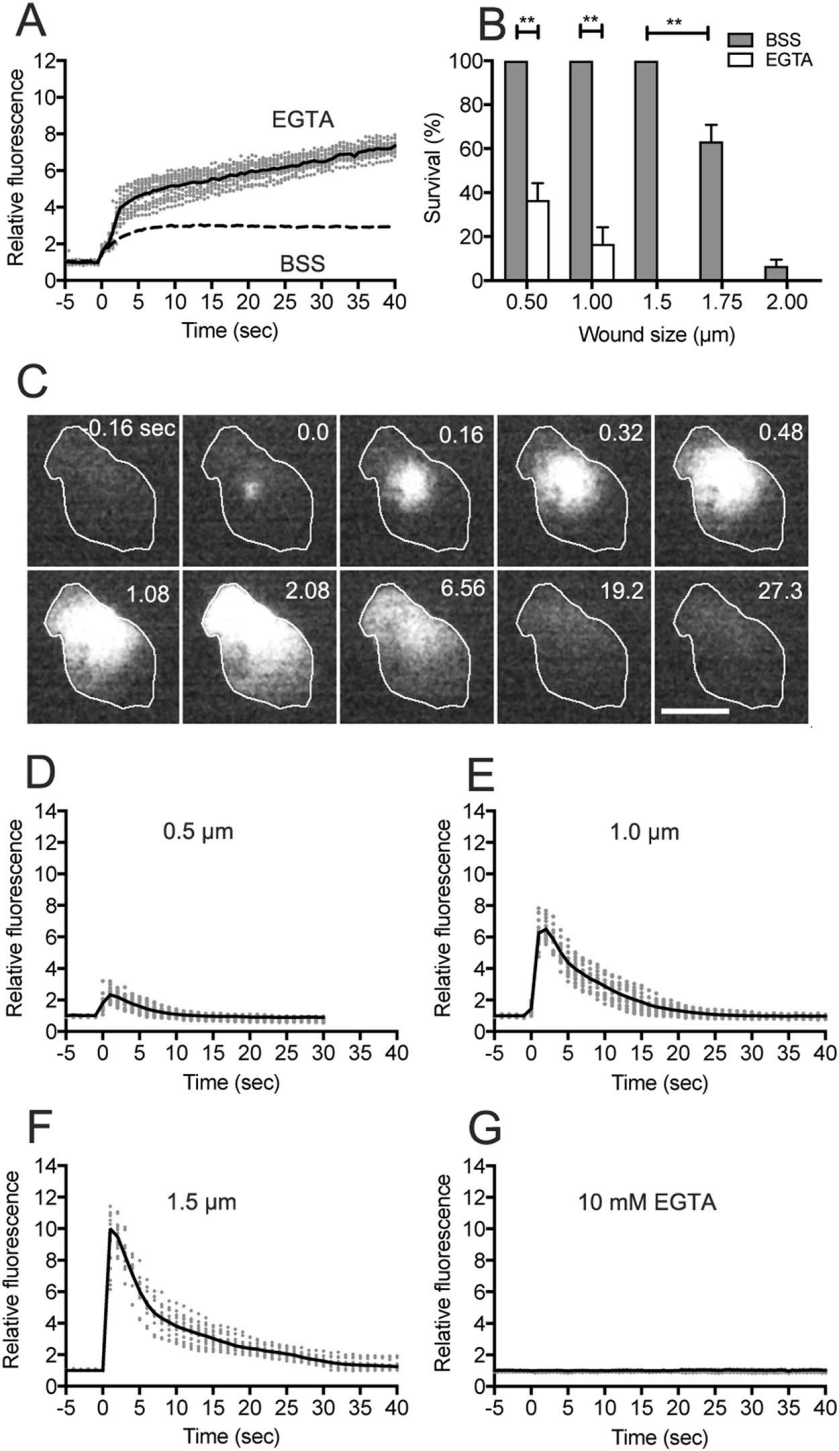
Ca^2+^ influx from the wound pore. (**A**) Time courses of PI influx in the presence (BSS) and absence (10 mM EGTA) of Ca^2+^ (1.0 µm wound). The solid line is an averaged line of 19 experiments in EGTA (gray dots) and the dashed line is an averaged line in BSS (from Fig. 2C). (**B**) Survival rate of different wound sizes in the presence (BSS) and absence (EGTA) of Ca^2+^. The number of cells that ruptured within 5 min after wounding was counted. Data are presented as mean ± SD (n = 20, 3 experiments, each). **P ≤ 0.0001. (**C**) A typical sequence of fluorescence images when a cell expressing Dd-GCaMP6s was wounded by laserporation. Note that the fluorescence began to increase at the wound site and spread over the cytoplasm. (**D**–**F**) Time courses of the fluorescence of Dd-GCaMP6s with different wound sizes (0.5, 1.0, and 1.5 µm). Each solid line shows an averaged line of 17 experiments (gray dots). (**G**) In the presence of 10 mM EGTA, the fluorescence of Dd-GCaMP6s did not increase after wounding (n = 20). Bar, 10 µm.

It has been reported that Ca^2+^ in the external medium is essential for wound repair^8,36,37^. We examined the effect of EGTA, a chelating agent of Ca^2+^, in the external medium. In the presence of EGTA, PI influx did not cease after wounding, whereas it ceased within a short time in BSS (Fig. 3A). Figure 3B shows the survival rate with different wound sizes in BSS and EGTA buffer. In the presence of EGTA, most of the cells ruptured within 5 min after wounding. Thus, Ca^2+^ in the external medium is essential for the wound repair of *Dictyostelium* cells.

Next, we examined whether Ca^2+^ entered the cytoplasm through the wound pore from the external medium. To visualize the cytosolic Ca^2+^ (Ca_i_^2+^), the cells expressing Dd-GCaMP6s, a Ca^2+^ sensor consisting of GFP, calmodulin, and a peptide sequence of myosin light chain kinase (M13), were wounded by laserporation. Figure 3C shows a typical time course of the fluorescence images of Ca^2+^ influx. The fluorescence immediately began to increase at the wound site and spread over the cytoplasm, suggesting that Ca^2+^ enters the cytoplasm through the wound pore. Figure 3D–F show the time courses of the fluorescence intensity with different wound sizes (0.5, 1.0, and 1.5 µm). With a larger wound size, the fluorescence increased. In the presence of EGTA, the fluorescence did not increase after wounding (Fig. 3G).

Together, the influx of Ca^2+^ through the wound pore contributes to the wound repair.

### Regulation of cytosolic Ca^2+^ after wounding

**Figure 4:**
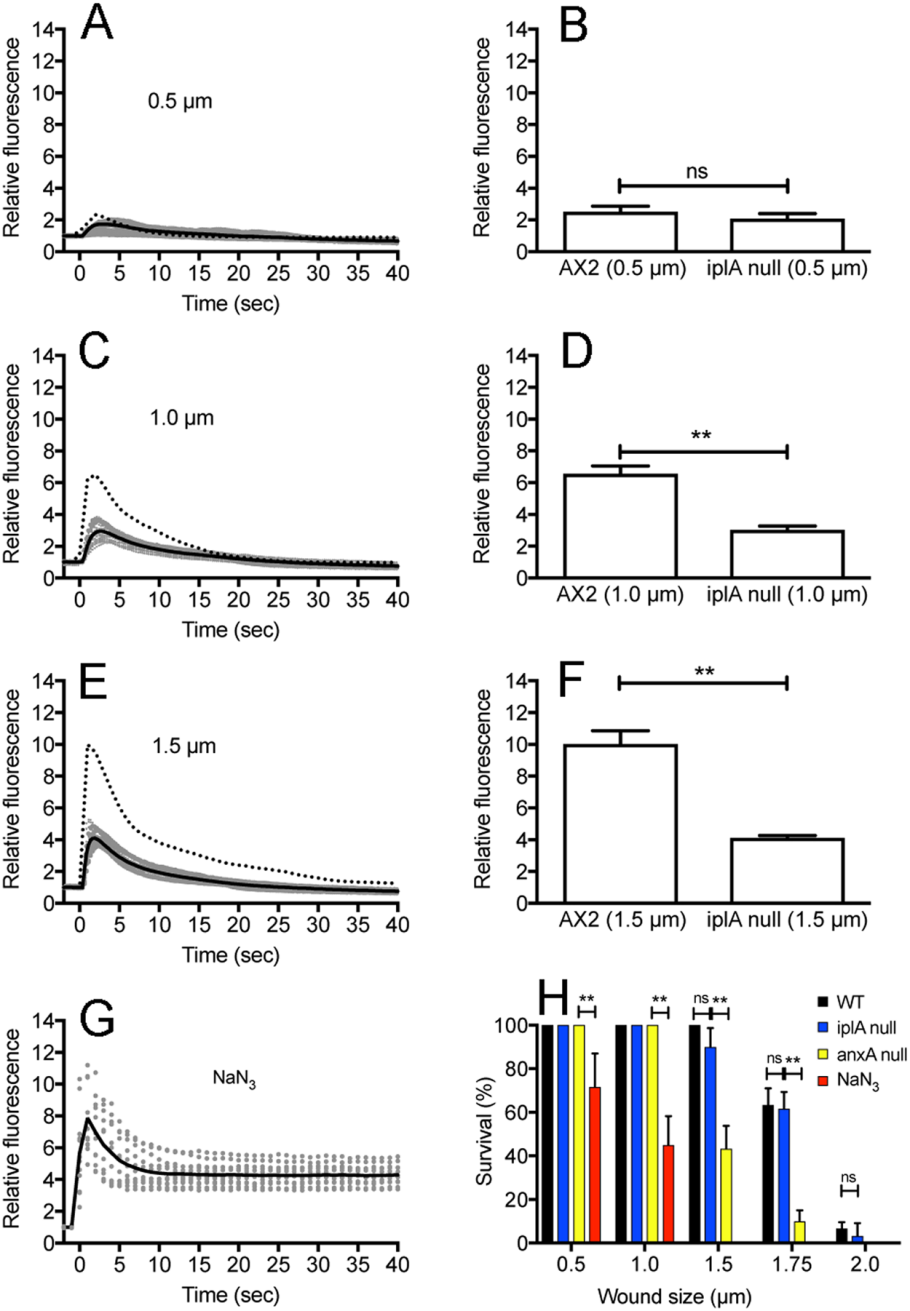
Regulation of cytosolic Ca^2+^ after wounding. (**A**,**C** and **E**). Time courses of Ca_i_^2+^ in iplA null cells expressing Dd-GCaMP6s with different wound sizes (0.5, 1.0, and 1.5 µm), respectively. The solid line is an averaged line of 15 experiments (gray dots) with each wound size and the dashed line is an averaged line in wild-type cells (from Fig. 3) for comparison. (**B**,**D** and **F**) Comparison of the highest fluorescence (peak) intensities between wild-type and iplA null cells with different wound sizes (0.5, 1.0, and 1.5 µm). Data are presented as mean ± SD. **P ≤ 0.0001; ns, not significant, P > 0.05. (**G**) Time courses of the Ca_i_^2+^ in the presence of NaN_3_ (1 µm wound). The solid line shows an averaged line of 15 experiments (gray dots). (**H**) Survival rate in the presence of NaN_3_, and those of mutants (iplA null and annexin C1 null cells) with different wound sizes. The number of cells that ruptured within 5 min after wounding was counted. Data are presented as mean ± SD (n = 20, 3 experiments, each). **P ≤ 0.0001; ns, not significant, P > 0.05.

The above results in the presence of EGTA indicate that only the wound without Ca^2+^ influx does not induce the elevation of Ca_i_^2+^. However, the influx of Ca^2+^ may induce Ca^2+^ release from intracellular Ca^2+^ stores via a calcium-induced calcium release (CICR) mechanism^38,39^, and this release may also contribute to the wound repair. To examine this possibility, iplA null cells expressing Dd-GCaMP6s were wounded. Inositol 1,4,5-triphosphate receptor-like protein A (iplA) has a homology to inositol trisphosphate receptors, which are mainly localized in intracellular membranous structures, such as the endoplasmic reticulum (ER), although this conclusion is still controversial^40^. These mutant cells show no increase in Ca_i_^2+^ after chemotactic stimulation^41^. Figure 4A,C and E show time courses of the Ca_i_^2+^ in iplA null cells with different wound sizes (0.5, 1.0, and 1.5 µm). The amplitude of the fluorescence was significantly reduced compared with that of wild-type cells with 1.0 and 1.5 µm wounding, suggesting that the Ca^2+^ release also partly contributes to the increase in Ca_i_^2+^ (Fig. 4D and F). However, following 0.5 µm wounding, there was no significant difference between wild-type and mutant cells (Fig. 4B). Presumably, a certain level of Ca_i_^2+^ may be required for activation of the CICR mechanism.

The elevated Ca_i_^2+^ shortly returned to a resting level, suggesting that Ca^2+^ is pumped out across the cell membrane or sequestered into Ca^2+^ stores. Next, we examined the effects of sodium azide, an inhibitor of ATP synthesis. In the presence of sodium azide, Ca_i_^2+^ did not return to a resting level after reaching a peak (Fig. 4G), suggesting that the energy is required for recovery to the resting level.

Figure 4H shows a summary of the survival rate of mutant cells and in the presence of sodium azide with different wound sizes. Sodium azide significantly reduced the survival rate, especially with a larger wound size, suggesting that the immediate decrease in Ca^2+^ is essential for cell survival. However, iplA null cells showed no significant difference in survival rate compared with wild-type cells, suggesting that the Ca^2+^ release by CICR is dispensable for wound repair.

### Annexin C1 accumulates at the wound site in a Ca^2+^-dependent manner

Annexin, a highly conserved ubiquitous family of Ca^2+^- and phospholipid-binding proteins, has been implicated in wound repair^15,31,42^. *Dictyostelium* has two annexin genes, annexin C1 (annexin VII or synexin) and annexin C2 (annexin I). Annexin C2 is expressed at approximately 35-fold lower levels compared to annexin C1, whereas both are expressed throughout the developmental stages^43^. There are two isoforms by alternate splicing, 51 kDa (full length) and 46 kDa of the single annexin C1 gene. Both can bind phosphatidylserine in a Ca^2+^-dependent manner^44,45^. Here we examined the dynamics of full length annexin C1 and annexin C2 during wound repair.

**Figure 5:**
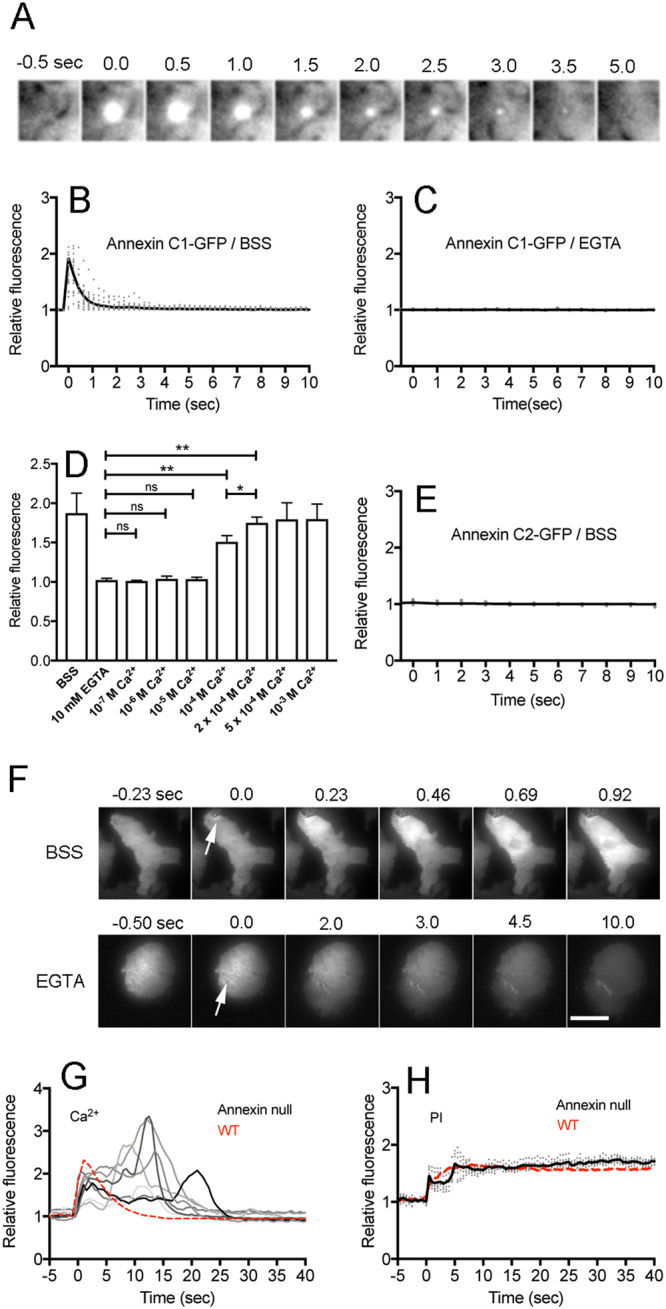
Annexin C1 accumulates at the wound site in a Ca^2+^-dependent manner. (**A**) A typical sequence of fluorescence images when cells expressing annexin C1-GFP were wounded by laserporation. Only a small area including the wound site is shown. (**B** and **C**) Time courses of fluorescence of annexin C1-GFP (B) in BSS and (**C**) in the presence of EGTA (0.5 µm wound, n = 15, each). (**D**) A dependency of the annexin C1-GFP accumulation on the free Ca^2+^ concentration in the external medium (0.5 µm wound). The peak intensities of annexin C1-GFP accumulation were plotted versus each Ca^2+^ concentration. Data are presented as mean ± SD (n = 15, each). *P ≤ 0.001; **P ≤ 0.0001; ns, not significant, P > 0.05. (**E**) Cells expressing annexin C2-GFP were wounded. Annexin C2-GFP did not accumulate at the wound site (n = 7). (**F**) A typical sequence of fluorescence images when a cell expressing annexin C1- GFP ruptured with 2.0 µm wound (arrows) in BSS (upper panels) and in the presence of EGTA (lower panels). Similar results were confirmed in 20 cells in each case. (**G**) Time courses of the fluorescence of Dd-GCaMP6s in annexin C1-null cells after wounding (0.5 µm wound). Each cell showed different irregular curves with multiple peaks (n = 7). The red dashed line is an averaged line in wild-type cells (from Fig. 3D) for comparison. (**H**) Time courses of PI influx in annexin C1 null cells (0.5 µm wound). The black solid line is an averaged line of 10 experiments (gray dots) and the red dashed line is an averaged line in wild-type cells (from Fig. 2B) for comparison. Bar, 10 µm.

Figure 5A shows a typical sequence of fluorescence images of a cell expressing annexin C1 (full length)-GFP after wounding, suggesting that it accumulated at the wound site immediately after laserporation. Figure 5B show time courses of the accumulation of GFP-annexin C1, indicating that it rapidly disappeared within approximately 1 sec (1.05 ± 0.26, n = 20) after wounding. In the presence of EGTA, annexin C1-GFP did not accumulated at the wound site (Fig. 5C). Figure 5D shows a dependency of annexin C1 accumulation on the external Ca^2+^ concentration, suggesting that annexin C1 significantly accumulated at free Ca^2+^ higher than 10^−4^ M. Annexin C2-GFP did not show any accumulation at the wound sites (Fig. 5E).

Interestingly, when the cell was ruptured with a larger wound (2 µm), the fluorescence of annexin C1-GFP rapidly spread from the wound site to the whole cell membrane within 1 sec (upper panels of Fig. 5F), whereas the fluorescence was limited at the wound site in successfully repaired cells (Fig. 5A). It has been previously reported that annexin C1 binds to the cell membrane at 10^−4^ M Ca^2+^ ^45^. A large quantity of Ca^2+^ influx seems to cause the binding of annexin C1 to the whole inner cell membrane. When the cell was ruptured in the presence of EGTA, the fluorescence of annexin C1-GFP gradually decreased, suggesting that it was released outside the cell without binding to the cell membrane (lower panels of Fig. 5F).

Next we examined wound repair in annexin C1-null cells. When annexin C1-null cells expressing Dd-GCaMP6s were wounded by laserporation (0.5 µm wound), each cell showed different irregular curves with multiple peaks, and Ca_i_^2+^ remained at a higher level (Fig. 5G) compared with wild-type cells (red dotted line). However, they finally returned to the resting level. PI influx was also examined in annexin C1-null cells, also showing irregular curves with multiple peaks in the initial phase (Fig. 5H) compared with wild-type cells (red dotted line). Annexin C1-null cells had a significantly reduced survival rate with larger but not small wound sizes (Fig. 4H).

Taken together, annexin C1 accumulates at the wound site depending on the influx of Ca^2+^ and partially contributes to the wound repair.

### Cells can respond to wounds multiple times

**Figure 6:**
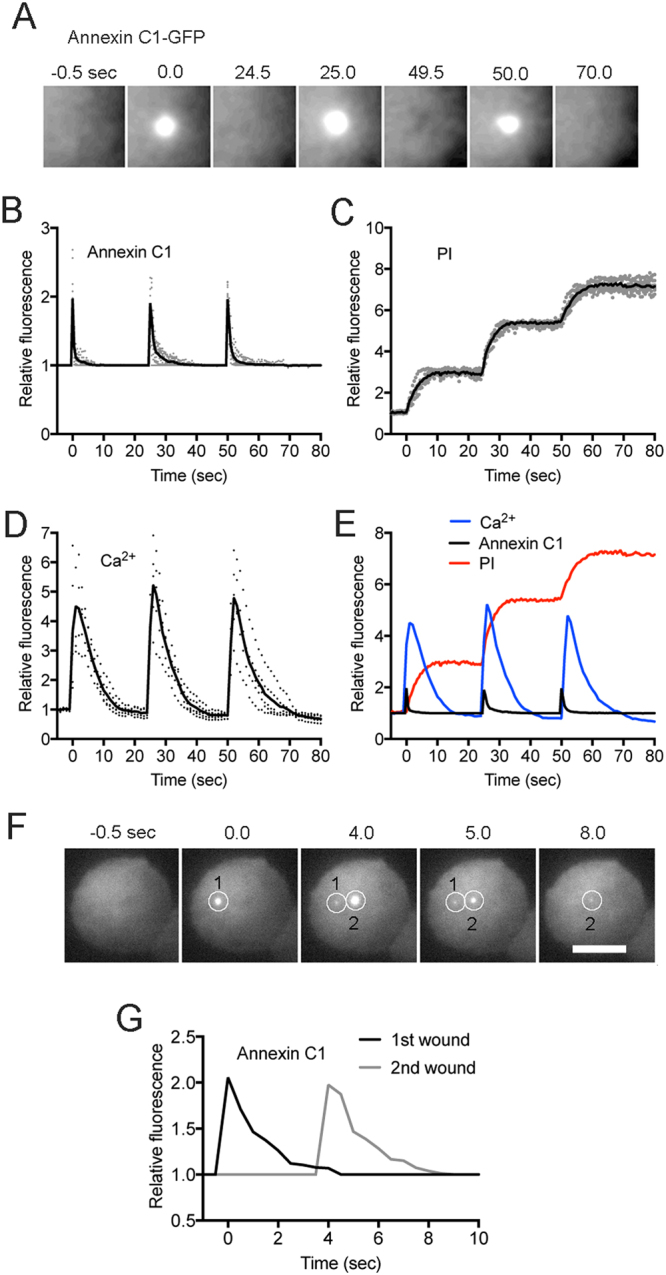
Cells can respond to wounds multiple times. (**A**) A typical sequence of fluorescence images when a cell expressing annexin C1-GFP was wounded 3 times at the same position. Only a small area including the wound site is shown. The laser was applied for 8 msec at 0, 25, and 50 sec, respectively. (**B**) Time courses of the fluorescence of annexin C1-GFP with 3 wounding events (n = 11). (**C**) Time courses of PI influx with 3 wounding events (n = 7). (**D**) Time courses of the fluorescence intensity of Dd-GCaMP6s with the 3 wound events (n = 5). (**E**) A summary of the averaged time courses of the three probes, annexin C1-GFP (black), PI (red), and Ca_i_^2+^ (blue), for comparison. (**F**) A typical sequence of fluorescence images when a cell expressing annexin C1-GFP was wounded 2 times at different positions (white circle 1 and 2). (**G**) Time courses of the fluorescence of annexin C1-GFP at 2 different positions in the cell shown in panel F. The laser was applied for 8 msec at 0 and 4 sec, respectively. Similar results were confirmed in 15 cells. All experiments in this figure were performed with the same wound size (0.5 µm).

It has been previously reported that the second wound at the same site reseals more rapidly than the initial wound in fibroblasts^11^. We examined multiple wounding at the same site in *Dictyostelium* cells. After wounding three times (0, 25, and 50 sec), annexin C1-GFP accumulated each time at almost the same level (Fig. 6A). Figure 6B shows the time courses of the annexin C1-GFP accumulation (n = 12). Figure 6C and D show the time courses of the influx of PI and the fluorescence intensities of Dd-GCaMP6s with three wounds, respectively (n = 8, each). Figure 6E shows a summary of time courses of the three probes, suggesting that *Dictyostelium* cells seem to have no such facilitated wound repair system in contrast to fibroblasts. If the cells had the second wound at different site, annexin C1-GFP showed a similar time course and amplitude as the initial wound (Fig. 6F and G).

Together, *Dictyostelium* cells show reproducible responses each time with multiple wounds.

## Discussion

In this study, we examined wound repair in *Dictyostelium* cells by using our novel laserporation method. Previous laserporation mainly uses an expensive high-power UV laser. Otherwise, a longer time exposure (1–5 sec) of the lower-power laser is used, but the longer exposure cannot be used for fast moving cells such as *Dictyostelium* cells and the exact wound timing may be obscure. In the present method, a short-term exposure (4–40 msec) of a low-power and low-cost laser is sufficient. As another merit, only the spot area of laser beam in the cell membrane is wounded, whereas the inside of the cell could also be wounded in previous methods. Since the laser used in the present study could not wound the cell membrane without the carbon coat, presumably the energy absorbed in the carbon creates a pore due to thermal energy (heat).

The size of a wound can be precisely regulated with our laserporation method. By changing wound sizes, we found that the cells could tolerate less than 2 µm wounds. Although the limit of the tolerable wound size has not been defined in other organisms, the wound sizes vary in other experiments: 25–50 µm for fruit fly embryos, 10–20 µm for frog eggs, 10–25 µm for sea urchin eggs, 0.5–2.5 µm for animal cultured cells, and 0.5 µm for yeast. Larger cells such as eggs and embryos seem to tolerate larger wound sizes. As one simple explanation, since larger cells have a larger volume, they could have a capacity to tolerate greater wounding without irreversible rupture than smaller cells. Another explanation is that larger cells have an additional mechanism, such as an actomyosin-based closing mechanism to repair larger wound, because the surface tension of larger cells is larger than that of smaller cells^32^. In fact, no accumulation of myosin II in wound sites has been reported in smaller cells such as fibroblasts, yeast, and *Dictyostelium* cells^9,32,46^, whereas larger cells, such as the fruit fly embryo and frog egg, form an actomyosin ring^19,37^.

After laserporation in cell expressing GFP-cAR1, the black area transiently expanded and then shrank (Fig. 1D and E), which might represent the dynamics of the wound hole. Similar transient expansion of the wound hole was observed in *Drosophila* embryo^6^. The transient expansion may be caused by the cell membrane tension immediately after wounding. However, the black area persisted approximately 8 sec until closing. In contrast, the PI influx experiments showed that PI influx ceased within 2–3 sec following a 0.5 µm wound. It is difficult to estimate a precise closing time from the Ca^2+^ influx experiments because the pumping out of Ca^2+^ or sequestering into Ca^2+^ stores should be taken into consideration. From the peak time in the presence of sodium azide (Fig. 4G), which inhibits almost all energy-dependent pumps, the actual ceasing time of Ca^2+^ influx is also estimated to be 2–3 sec, which is consistent with the results of PI influx experiments, but inconsistent with the 8 sec in the results of the GFP-cAR1 experiments. Presumably, up to 8 sec, the wound area remains uneven with the surrounding cell membrane, excluding the GFP-cAR1 although the wound pore functionally closes within 2–3 sec. Some intracellular small vesicles may fuse to the wound cell membrane, which may exclude GFP-cAR1.

In the presence of EGTA, PI influx continued at slower rate after an immediate increase, whereas it ceased in BSS (Fig. 3A). This observation suggests that wound repair without Ca^2+^ influx partially functions but it is incomplete. There seems to be at least two mechanisms in wound repair. One is Ca^2+^ influx-dependent, which evokes the recruitment of annexin C1, potentially leading to the vesicles accumulation to reseal the wound pore. The other is a Ca^2+^ influx-independent pathway, such as rab-5 and rab-11^47^.

The present and previous^32^ studies showed that at least 0.1 mM extracellular Ca^2+^ is required for wound repair for *Dictyostelium* cells. However, the soil where these cells are normally found may not have such high levels of free Ca^2+^, which also supports the existence of Ca^2+^ influx-independent pathway. In addition, it is very interesting from the aspect to evolution that a Ca^2+^-dependent primordial repair mechanism exists in cells that may not normally be exposed to such levels of Ca^2+^.

The present results clarify why annexin C1-GFP locally accumulates only at the wound site. When the cells ruptured with a larger wound, annexin C1-GFP bound to the whole cell membrane (Fig. 5F). *In vitro*, annexin C1 binds to the cell membrane at 10^−4^ M Ca^2+^, but not in the presence of EGTA^45^. Because annexin C1-GFP accumulates in the wound site at concentrations higher than 10^−4^ M Ca^2+^ in the external medium (Fig. 5D), the concentration of Ca^2+^ must locally increase to levels higher than 10^−4^ M around the wound site. The fluorescence of annexin C1-GFP increased only at the wound site, but not at the other cell membrane, whereas Ca_i_^2+^ spread and increased throughout the cytoplasm (Fig. 2A). Presumably, this level of Ca_i_^2+^ will not cause the binding of annexin C1-GFP to the other cell membrane. If the level of Ca_i_^2+^ exceeds 10^−4^ M, annexin C1 will bind to the entire cell membrane.

Based on the experiments using iplA null mutants (Fig. 4), the source of Ca_i_^2+^ elevation is not only Ca^2+^ influx but also Ca^2+^ release from Ca^2+^ stores. The present data clearly shows the existence of the CICR system in *Dictyostelium* cells, as suggested previously^39^. The observation that the survival rate after wounding was not reduced in iplA null cells suggests that the additional increase in Ca_i_^2+^ by CICR followed by Ca^2+^ influx is dispensable for wound repair. Moreover, the elevation of Ca^2+^ throughout the cytosol also may not be necessary for local wound repair.

The experiments using sodium azide suggest that energy is required to return the Ca_i_^2+^ to the resting level, which is essential for cell survival. The responsible Ca^2+^ stores and membrane pump remain to be determined in the future. The present wound method can serve also as a powerful tool to investigate cytosolic Ca^2+^ homeostasis.

*Dictyostelium* cells can survive after microneedle cutting as shown in previous experiments^32–34^. The wound size by microneedle cutting could be much larger than the limit of wound size examined in the present study (less than 2 µm). However, for microneedle cutting, the cell is gently pressed with a microneedle and then cut by swiftly pulling it. This pressing may fuse the upper and lower cell membranes; thereby the actual wound pore may be smaller than the apparent.

In our previous experiments by microneedle cutting, actin begins to accumulate at approximately 2–3 sec and peaks at about 6–10 sec after wounding^32^, which is significantly slower than the timing of annexin C1 accumulation. In future, the connection between Ca^2+^ influx, annexin C1 accumulation, and cytoskeletal accumulation must be investigated.

The reaction of wound repair is attractive for cell biology because it is inducible, dynamic, and repeatable, involving membrane trafficking, membrane fusion, and dynamics of the cytoskeleton^48^. *Dictyostelium* has been recognized as a model organism for research of cell biology, such as cytokinesis, phagocytosis, cell migration, and chemotaxis. There are many available resources of knockout mutants of cytoskeletal proteins, intracellular signals, and membrane traffic proteins, most of which are almost the same as those found in higher animal cells. Since the genome of *Dictyostelium* is haploid, it is easy to generate knockout mutants and expression of tag genes. It is also valuable to know the evolution of wound repair mechanism.

We believe that *Dictyostelium* cells will become a new member of excellent model organisms for research in wound repair.

## Methods

### Cell culture

*Dictyostelium discoideum* cells (AX2) were cultured in plastic dishes at 22 °C in HL5 medium (1.3% bacteriological peptone, 0.75% yeast extract, 85.5 mM D-glucose, 3.5 mM Na_2_HPO_4_. 12H_2_O, and 3.5 mM KH_2_PO_4_, pH 6.3), as previously described^49^. The cells were transformed with extrachromosomal vectors for the expression of GFP-cAR1, Dd-GCaMP6s, annexin C2-GFP, or annexin C1-GFP by electroporation as previously described^50^. The transformed cells were selected in HL5 medium supplemented with 10 µg/ml of G418 (Sigma). For the wound experiments, HL5 medium was replaced with BSS (3 mM CaCl_2_, 10 mM KCl, 10 mM NaCl, 3 mM MES, pH 6.3), and the cells were incubated in the same solution for 4–5 hrs.

### Plasmid construction

The vectors of annexin C1-GFP and annexin C2-GFP were newly constructed. Each gene was amplified from cDNA library (a gift from Dr. D. Robinson) and inserted into the pA15GFP expression vector including a G418 resistance gene. Dd-GCaMP6s was used as a Ca^2+^ probe, in which the codon usage in the original GCaMP6s^51^ was optimized for *Dictyostelium*. To generate the knockout construct of annexin C1, annexin C1 fragment was subcloned outside of two loxP sites in pLRBLP. The left arm (nucleotides 50–600) and right arm (nucleotides 619–1196) of annexin C1 gene were amplified from the cDNA by PCR using the following primer sets with restriction enzyme sites (underscore): 5′-ATGTCGACCACAACAAGGTTATCCACCACAACAAGGCT-3′ (SalI) and 5′-ATATCGATGTGAATTTGTTCAACATCGAAATGAGCTGG-3′ (ClaI) (for the left arm of the KO construct); and 5′-ATGGATCCGGTACCAACGAGAACACTATAATTGAAATTTTAG-3′ (BamHI) and 5′-ATACTAGTGCTAATGAATTCTTGAAGAGAGTTGAATAAGC-3′ (SpeI) (for the right arm of the KO construct). The amplified left arm fragments were subcloned between SalI and ClaI sites lying upstream of N-terminal loxP site in pLRBLP. The right arm fragments were subsequently subcloned between BamH1 and SpeI sites lying downstream of C-terminal loxP site. After transformation with the knockout construct, cells were selected in HL5 medium containing 10 µg/ml of blasticidin S hydrochloride (Wako).

### Carbon coating and chamber preparation

The surface of the coverslip of a glass-bottom chamber was coated with carbon by vapor deposition using a vacuum evaporator (JEOL, JEE-400). The thickness of the carbon layer was approximately 20 nm. To make the surface hydrophilic, the surface of the carbon-coated coverslip was activated with a plasma treatment. The chamber was sterilized with 70% ethanol and dried if necessary. The cells were settled on the surface of the carbon-coated coverslip, and they were slightly compressed with agarose block (1.5%, dissolved in BSS, 1 mm thick) to observe the ventral cell surface^52^.

### Laserporation

The fluorescence images of cells expressing GFP-proteins were observed under a total internal reflection fluorescence microscope (TIRF, based on IX71 microscope, Olympus) as previously described^53^. For laserporation, a nanosecond-pulsed laser (FDSS532-Q, CryLas) beam was directed toward the sample using a dichroic mirror through the TIRF microscope as previously described^35^. The laser beam (wavelength, 532 nm) was operated at a 15 mW output power and pulse width of 1 nsec and attenuated to 1/300 via passage through several neutral density filters. A 60 × (PLAPON60XOTIRFM, Olympus, NA = 1.45) objective was used to focus the laser beam on the surface of the carbon-coat. When the laser beam was applied by properly adjusting the focus, the carbon coat was peeled off, appearing as a small white spot (Fig. 1C). The x-y position of the laser spot was fixed in the center of the microscopic field. The position of the cells was moved using a piezo-motorized x-y stage on the microscope. The duration of the laser beam application was set at 4–40 msec and controlled by a shutter. The wound size was regulated by changing the laser power or the size of pinhole, which was inserted on the pathway between the laser and dichroic mirror. Time-lapse fluorescence images were acquired with 50–100 msec exposure times at 200–500 msec intervals using a cooled CCD camera (Orca ER, Hamamatsu Photonics).

### Scanning electron microscopy (SEM)

The laser beam was applied on the surface of carbon-coated coverslip to generate various-sized white spots. After acquiring the images of white spots under a light microscope, the same coverslip was directly attached to the SEM mount folder with double-sided carbon sticky tapes to remove negative charges. The coverslip was observed under a scanning electron microscope (JEOL, JSM-6360LA).

### Measurement of the influx of propidium iodide and Ca^2+^

To characterize the opening and closing of wound pores, 0.15 mg/ml of propidium iodide (PI, Sigma) was included in the external medium (BSS). When PI enters the cytoplasm, it emits red fluorescence by an argon laser (488 nm) under TIRF microscopy.

To monitor the cytosolic Ca^2+^, cells expressing Dd-GCaMP6s were wound by laserporation. This Ca^2+^ probe emits green fluorescence (>515 nm) under illumination by an argon laser (488 nm), depending on the Ca^2+^ concentration.

Sodium azide (Wako) was dissolved in distilled water to generate a 100 mM stock solution. A small aliquot of the stock solution was applied to the surface of the agarose blocks to make the indicated final concentration (5 mM). Cells were incubated with sodium azide from 30 min before the wound experiments.

### Image analysis

The acquired images were analyzed with Image J software (http://rsbweb.nih.gov/ij/). The time course of relative fluorescence intensity at the wound region was calculated by dividing the fluorescence intensity at the wound site by that of the other cytoplasmic region after each value was subtracted from the background. The graphs of the time course of the relative fluorescence intensity were created using GraphPad Prism 7 (GraphPad Inc., USA) after calculation using Microsoft Excel.

To examine the half time of PI influx to reach a maximum after wounding, the time course of relative fluorescence intensity (Fr) was fitted to following formula (1) for a single exponential rise to maximum:

1 $${\rm{Fr}}={\rm{a}}[1-\exp (-{\rm{bt}})]+{\rm{c}}$$

where a is amplitude of exponential, b is rate constant, t is time (sec), and c is constant. The half time (t_1/2_) is calculated using the following formula (2):

2 $${{\rm{t}}}_{1/2}=-\,\mathrm{ln}(0.5)/{\rm{b}}$$

### Ca-EGTA buffer

To formulate medium containing the indicated free Ca^2+^, a Ca-EGTA buffer (10 mM KCl, 10 mM NaCl, 3 mM MES, 10 mM EGTA and an appropriate concentration of CaCl_2_, pH 6.3) was used. The concentration of CaCl_2_ in the Ca-EGTA buffer was calculated by Ca-EGTA Calculator v1.3 (http://maxchelator.stanford.edu/CaEGTA-TS.htm). The agarose block for the agar-overlay was made by dissolving 1.5% agarose in Ca-EGTA buffer or BSS as previously described^52^.

### Statistical analysis

Statistical analysis was performed using GraphPad Prism 7. Data are presented as the mean ± SD and analyzed by one-way ANOVA with Tukey’s multiple comparison test.

### Data availability

All relevant data are available from the authors on reasonable request.
